# Multi-level tuberculosis of the spine identified by 18 F-FDG-PET/CT and concomitant urogenital tuberculosis: a case report from the spinal TB X cohort

**DOI:** 10.1007/s15010-024-02327-5

**Published:** 2024-06-19

**Authors:** Julian Scherer, Sandra L. Mukasa, Karen Wolmarans, Reto Guler, Tessa Kotze, Taeksun Song, Robert Dunn, Maritz Laubscher, Hans-Christoph Pape, Michael Held, Friedrich Thienemann

**Affiliations:** 1 General Medicine & Global Health (GMGH), Department of Medicine and Orthopaedic Research Unit (ORU), Division of Orthopaedic Surgery, Faculty of Health Science, University of Cape Town, Cape Town, South Africa; 2 Department of Traumatology, University Hospital Zurich, University of Zurich, Zurich, Switzerland; 3 General Medicine & Global Health (GMGH), Department of Medicine, Faculty of Health Science, University of Cape Town, Cape Town, South Africa; 4 Institute of Infectious Diseases and Molecular Medicine (IDM), Department of Pathology, Division of Immunology, Faculty of Health Sciences, University of Cape Town, Cape Town, South Africa; 5 International Centre for Genetic Engineering and Biotechnology (ICGEB), Cape Town Component, Cape Town, South Africa; 6 Department of Medicine, CUBIC, PETCT, University of Cape Town, Cape Town, South Africa; 7 Institute of Infectious Diseases and Molecular Medicine (IDM), Department of Pathology, Division of Medical Microbiology, Faculty of Health Sciences, University of Cape Town, Cape Town, South Africa; 8 Orthopaedic Research Unit (ORU), Division of Orthopaedic Surgery, Faculty of Health Science, University of Cape Town, Cape Town, South Africa; 9 Department of Internal Medicine, University Hospital Zurich, University of Zurich, Zurich, Switzerland

**Keywords:** TB, Spondylodiscitis, Tuberculous spondylodiscitis, Pott’s disease, Spine, Infection, PET/CT, 18F-FDG-PET/CT

## Abstract

**Background:**

Tuberculosis (TB) is caused by *Mycobacterium tuberculosis* (*Mtb*) and typically infects the lungs. However, extrapulmonary forms of TB can be found in approximately 20% of cases. It is suggested, that up to 10% of extrapulmonary TB affects the musculoskeletal system, in which spinal elements (spinal tuberculosis, STB) are involved in approximately 50% of the cases. STB is a debilitating disease with nonspecific symptoms and diagnosis is often delayed for months to years. In our Spinal TB X Cohort, we aim to describe the clinical phenotype of STB using whole-body 18 F-fluorodeoxyglucose positron emission tomography computed tomography (PET/CT) and to identify a specific gene expression profile for the different stages of dissemination on PET/CT. Here we report on the first patient recruited into our cohort who underwent PET/CT before treatment initiation, at 6-months and at 12-months - time of TB treatment completion.

**Case presentation:**

A 27-year-old immunocompetent male presented with severe thoracolumbar back pain for 9 months with severe antalgic gait and night sweats. Magnetic resonance imaging (MRI) of the whole spine revealed multilevel spinal disease (T5/6, T11/12, L3/4) in keeping with STB. After informed consent and recruitment into the Spinal TB X Cohort, the patient underwent PET/CT as per protocol, which revealed isolated multilevel STB (T4-7, T11/12, L3/4) with no concomitant lung or urogenital lesion. However, sputum and urine were Xpert MTB/RIF Ultra positive and *Mtb* was cultured from the urine sample. CT-guided biopsy of the T11/12 lesion confirmed drug-sensitive *Mtb* on Xpert MTB/RIF Ultra and the patient was started on TB treatment according to local guidelines for 12 months. The 6-month follow-up PET/CT revealed new and existing spinal lesions with increased FDG-uptake despite significant improvement of clinical features and laboratory markers. After 9 months of treatment, the patient developed an acute urethral stricture, most likely due to urogenital TB, and a suprapubic catheter was inserted. The 12-month PET/CT showed significantly decreased PET/CT values of all lesions, however, significant persistent spinal inflammation was present at the end of TB treatment. Clinically, the patient was considered cured by the TB control program and currently awaits urethroplasty.

**Conclusions:**

In our case, PET/CT emerged as a valuable imaging modality for the initial assessment, surpassing MRI by revealing more comprehensive extensive disease. Subsequent PET/CT scans at 6-month uncovered new lesions and increased inflammation in existing ones, while by the end of TB treatment, all lesions exhibited improvement. However, the interpretation of FDG avidity remains ambiguous, whether it correlates with active infection and viable Mtb. or fibro- and osteoblast activity indicative of the healing process. Additionally, the absence of extraspinal TB lesions on PET/CT despite positive microbiology from sputum and urine maybe explained by paucibacillary, subclinical infection of extraspinal organs. The Spinal TB X Cohort endeavours to shed light on whole-body imaging patterns at diagnosis, their evolution midway through TB treatment, and upon treatment completion. Ultimately, this study aims to advance our understanding of the biology of this complex disease.

## Introduction

Tuberculosis (TB) is one of the most common infections worldwide and one of the leading causes of death. Every year about 10 million people fall ill with TB and 1.6 million died in 2021 [1]. TB is caused by *Mycobacterium tuberculosis (Mtb*) and typically infects the lungs, but other organs and anatomical sites can be affected (extrapulmonary TB) [2]. Extrapulmonary TB accounts for almost 20% of TB cases worldwide [3]. Literature suggests that approximately 10% of extrapulmonary TB affects the musculoskeletal system, and in about 50% of those, spinal elements are involved [4–6]. Spinal TB (STB, spondylitis or spondylodiscitis caused by *Mtb*) is often referred to as Pott`s Disease and can result in back pain, fever, night sweats, loss of weight, neurological signs and neurogenic symptoms such as bowel and urinary incontinence. Initial symptoms are often nonspecific and slowly progressive, with common delay from symptom onset to diagnosis, sometimes even several years [7, 8]. Approximately 50% of STB cases have either active pulmonary TB disease or have previously suffered from pulmonary TB disease, and immunocompromised patients or people living with HIV are more susceptible to develop extrapulmonary and disseminated forms of TB [9–11]. 

Diagnostic workup for a suspected case of STB includes a computed tomography (CT) scan of the spine and magnetic resonance imaging (MRI), the latter is considered “imaging gold standard”. Tissue sampling is required for a definite diagnosis and requires invasive procedures (CT-guided biopsy or open surgery) [12]. 

In our Spinal TB X cohort (ClinicalTrials.gov NCT05610098), we aim to describe the clinical phenotype of STB using whole-body 18 F-fluorodeoxyglucose positron emission tomography computed tomography (PET/CT) and to identify a specific gene expression profile for the different stages of dissemination. A blood-based test for STB would lead to earlier diagnosis and treatment in all settings globally and improve treatment outcome of this devastating disease.

Here we report on a 27-year-old, immunocompetent man included into our cohort study with microbiologically confirmed multilevel STB and concomitant urogenital TB. The initial PET/CT revealed more extensive lesions compared to MRI. The 6-month follow-up PET/CT showed radiological deterioration of spinal lesions and the patient developed an acute urethral stricture after 9 months under TB treatment. The patient was considered cured at the final 12-month PET/CT and discharged from the TB control program despite persistent spinal inflammation and thus may be of risk of post tuberculosis diseases.

## Case report

### Patient history and clinical exam

A 27-year-old male truck driver presented to a secondary level orthopaedic department in Cape Town complaining of a 9-month history of worsening thoracic and lumbar back pain. The patient reported no previous history of TB or trauma but complained of night sweats. Other typical symptoms of TB such as unintentional weight loss, fever and cough were absent. The patient reported no previous medical conditions.

On clinical examination, the patient exhibited an antalgic gait due to pain, with no neurological deficits or motor dysfunction and normal reflexes. There were no neurogenic symptoms (urinary or bowel symptoms) present. General examination, including abdominal, respiratory, cardiac, and lymphatic systems, appeared normal. The specific spinal examination revealed gibbus formation over T11/12. The patient reported an overall pain visual analogous scale (pVAS) of 7/10, with mechanical and axial loading pain and paravertebral tenderness. While the patient was able to ambulate, it was only possible with an assistive device, due to excruciating pain. Baseline and follow-up blood investigations are summarized in Table 1.

**Table 1:** Baseline and PET/CT 2/3 blood-investigations

Investigation	Result (baseline)	Results (PET/CT 2)	Results (PET/CT 3)	Reference values
HIV ELISA	Negative	N/A	N/A	Negative
CD4 count (cells/µL):	N/A	N/A	N/A	332–1642
Viral Load (IU/L)	N/A	N/A	N/A	Lower than detectable
White Cell Count (x10^9^/L):	5.96	6.19	8.83	3.92–10.40
Monocytes (x10^9^/L):	0.83*	0.37	0.50	0.30–0.80
Eosinophils (x10^9^/L):	0.04	0.23	0.21	0.00-0.95
Basophils (x10^9^/L):	0.05	0.05	0.06	0.00-0.10
Lymphocytes (x10^9^/L):	1.78	1.96	2.21	1.40–4.20
Neutrophils (x10^9^/L):	3.24	3.55	5.90	1.60–6.98
Haemoglobin (g/dL):	10.8*	12.9*	14.0	13–17
Platelets (x10^9^/L):	438*	252	270	171–388
Creatinine (µmol/L):	112*	77	86	64–104
ALT (U/L):	30	29	107*	10–40
CRP (mg/L):	51*	15*	12*	< 10
HbA_1_C (%):	5.3	N/A	N/A	< 6.5

ALT: Alanine Transferase; CRP: C-reactive Protein, HbA1C: Hemoglobin A1C, *pathological value

### Imaging

**Fig. 1:**
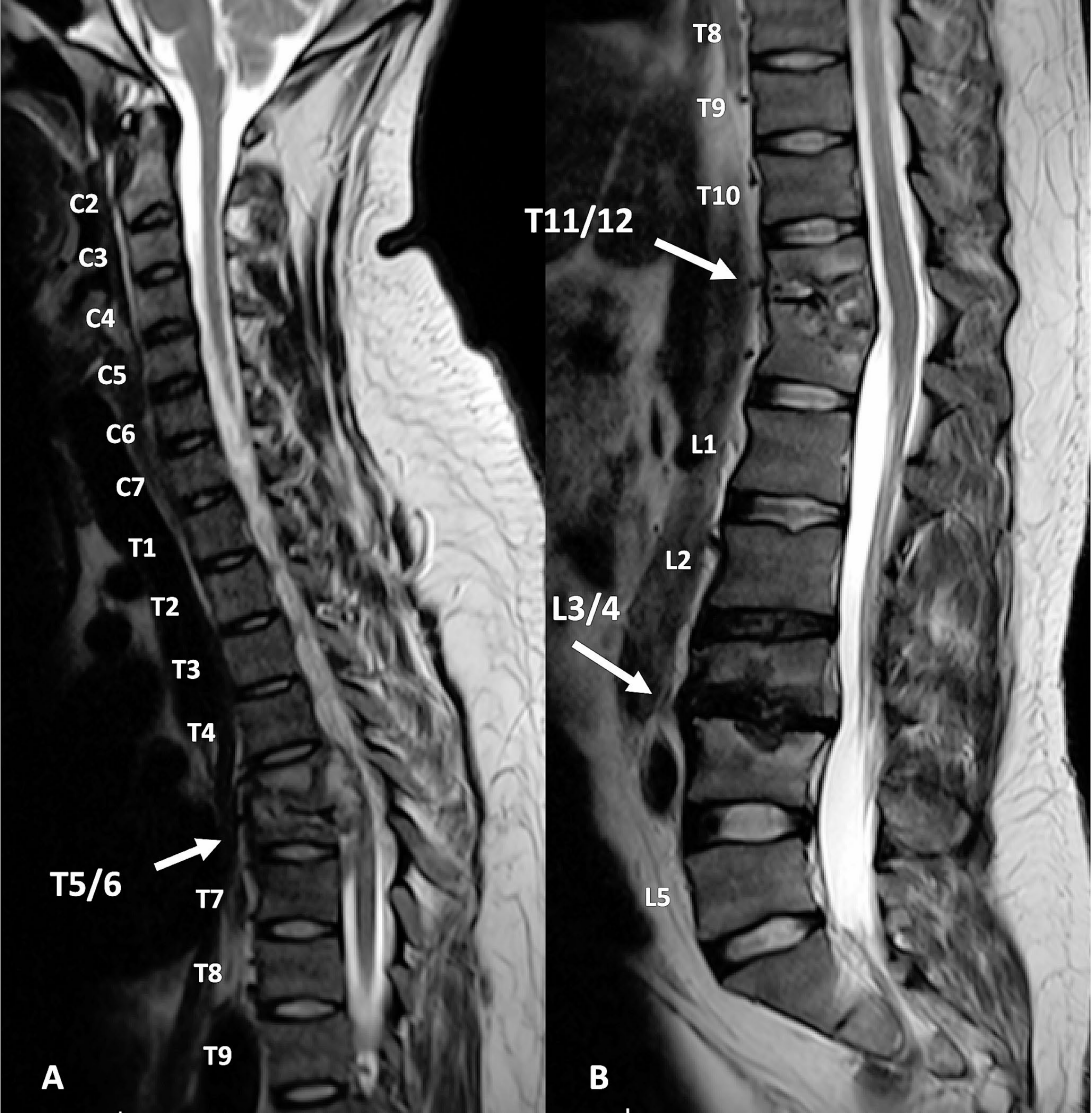
**A**: T2-weighted sagittal MRI of the upper vertebral column showing T5/6 endplate destruction with mild kyphotic deformity. Intervertebral disc space integrity lost, but low signal disc still visualized. Heterogenous T2 signal soft tissue effacing the anterior CSF space and impressing the anterior cord. **B**: T2-weighted sagittal MRI of the lower vertebral column showing T11/12 endplate destruction with moderate kyphotic deformity. Intervertebral disc space integrity lost, but low signal disc still relatively maintained. Heterogenous T2 signal soft tissue minimally effacing the anterior CSF space and without cord compromise. L3/4: Endplate destruction with moderate kyphotic deformity. Intervertebral disc space integrity lost, but low signal disc still relatively maintained. Heterogenous T2 signal soft tissue minimally effacing the anterior CSF space without cauda equina compromise

Subsequent MRI of the whole spine revealed high suspicion of STB, showing multilevel thoracolumbar spondylitis with endplate destruction and relative disc sparing of T5/6, T11/12 and L3/4, but no cord signal changes or psoas abscess (Fig. 1).

**Fig. 2:**
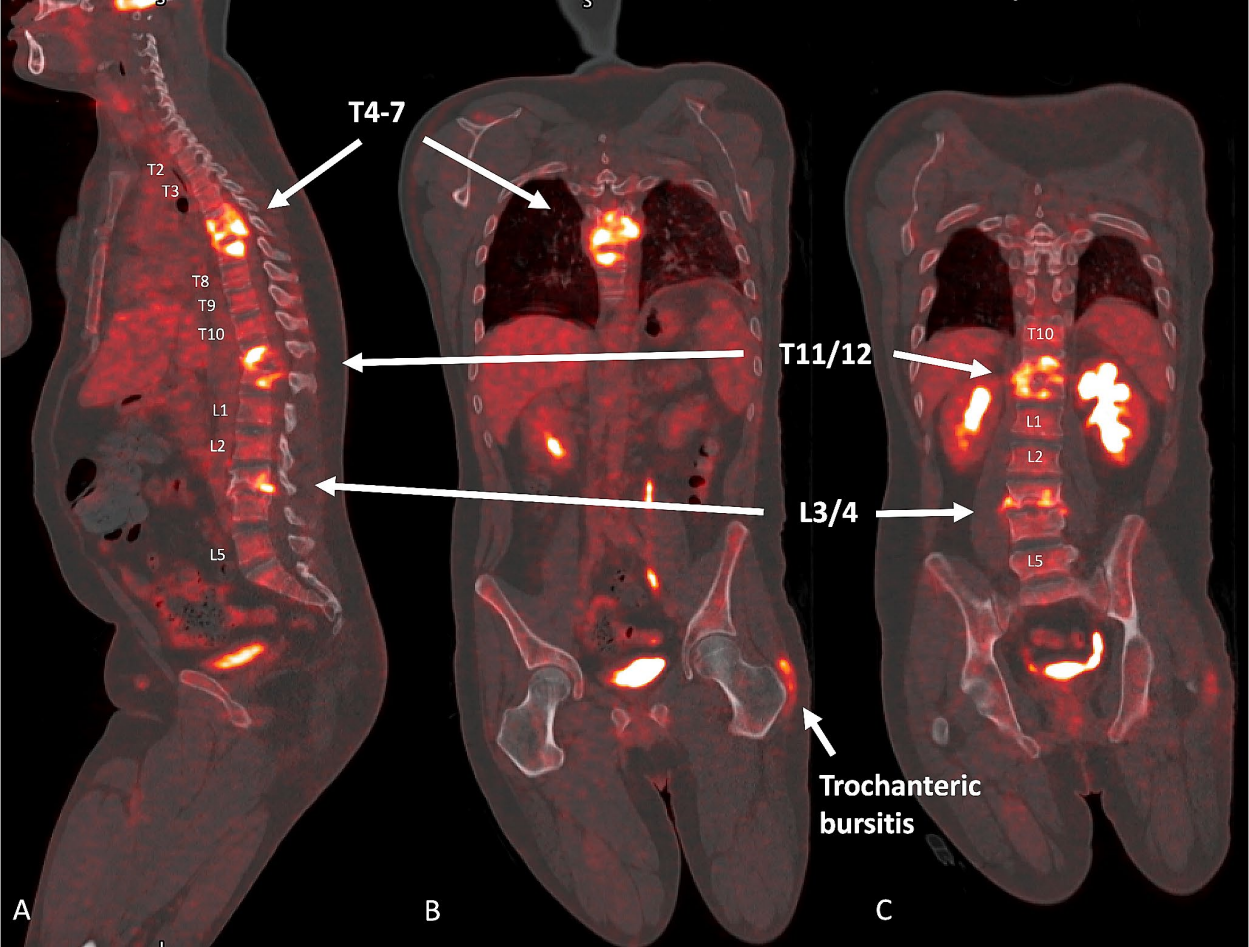
PET/CT before treatment initiation **A**: Whole-body 18-FDG-PET/CT sagittal view. **B**: Whole-body 18-FDG-PET/CT coronal view thoracic spine. **C**: Whole-body 18-FDG-PET/CT coronal view thoracolumbar spine

The baseline whole-body (from the base of skull to mid-thigh) PET/CT scan, conducted prior to CT-guided biopsy and treatment initiation using a Siemens Biograph mCT, demonstrated FDG avid lesions in T4-7, T11/12 and L3/4, along with left trochanteric bursitis, most likely due to prolonged left-sided positioning of the patient during hospitalization (Fig. 2).

**Fig. 3:**
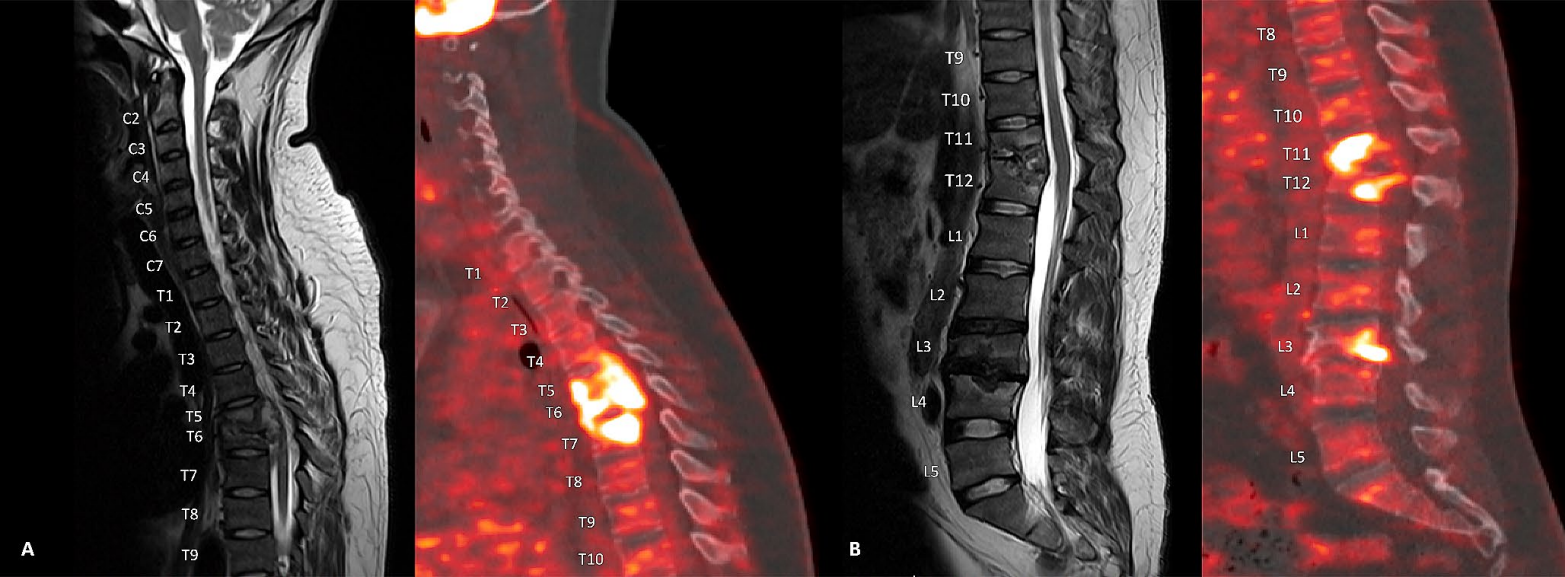
**A**: Upper thoracic lesion on MRI and 18-FDG-PET/CT on sagittal plane. **B**: Thoracolumbar lesions on MRI and 18-FDG-PET/CT on sagittal plane

**Fig. 4:**
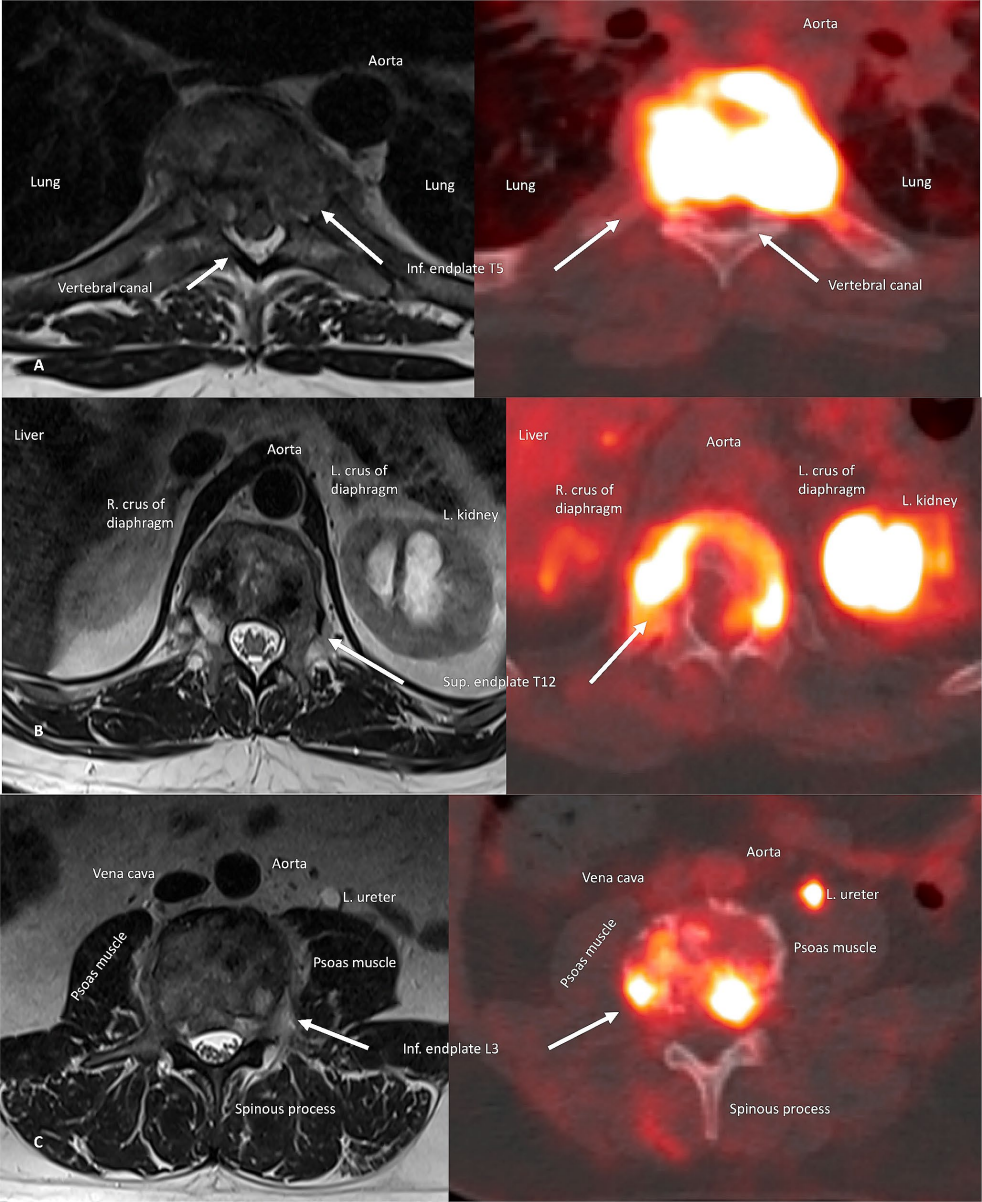
**A**: T5/6 lesion on MRI and 18-FDG-PET/CT on axial plane. **B**: T11/12 lesion on MRI and 18-FDG-PET/CT on axial plane. **C**: L3/4 lesion on MRI and 18-FDG-PET/CT on axial plane

The radiological findings of the initial PET/CT are outlined in Table 2. Figures 3 and 4 show the visual comparison of MRI and PET/CT at the different sites of spinal involvement.

**Table 2:** PET/CT findings before treatment initiation

Region	Findings	SUVmax	Comment
Neck	Linear increased uptake posterior to the left sternocleidomastoid muscle.	4.72	On CT there is no discrete lesions seen. Uptake localises to vascular structure. No abnormal uptake in the thyroid gland or cervical nodes.
Thorax	No abnormal uptake seen in the lung parenchyma and mediastinum.	N/A	On CT the heart, great vessels, trachea and oesophagus have a normal appearance. On CT the pulmonary parenchyma is clear with no infiltrates. No effusions. No pneumothorax.
Abdomen	Focal increased uptake seen in the right lateral abdominal wall. On CT there is a 12 × 13 mm lesion between the muscles of the right abdominal wall.	5.87	No abnormal uptake is seen in the liver, spleen, adrenal glands or retroperitoneal. On CT liver, spleen, pancreas, adrenals and kidneys have a normal appearance. No free fluid or gas is seen in the abdomen and pelvis. Vascular structures appear normal. The small focal lesion with increased SUV uptake was clinically investigated and found to be most likely due to long-term bed rest.
Skeletal	Focal uptake in the left scapula.	3.56	No destructive bony changes on CT.
	T4-7	17.39	On CT there is marked destruction of the adjacent endplates of T4-7 with lytic destruction of the bodies.
	T11/12	17.57	On CT there is marked destruction of the adjacent endplates of T5/6 with lytic destruction of the bodies.
	L3/4	11.77	On CT there is marked destruction of the adjacent endplates of T5/6 with lytic destruction of the bodies.
Incidental	Increased uptake seen in the soft tissue lateral to the greater trochanter of the left hip	7.65	This is likely inflammatory due to long-tern immobilization.

T: thoracic spine; L: lumbar spine; SUVmax: maximum standardized uptake value

**Fig. 5:**
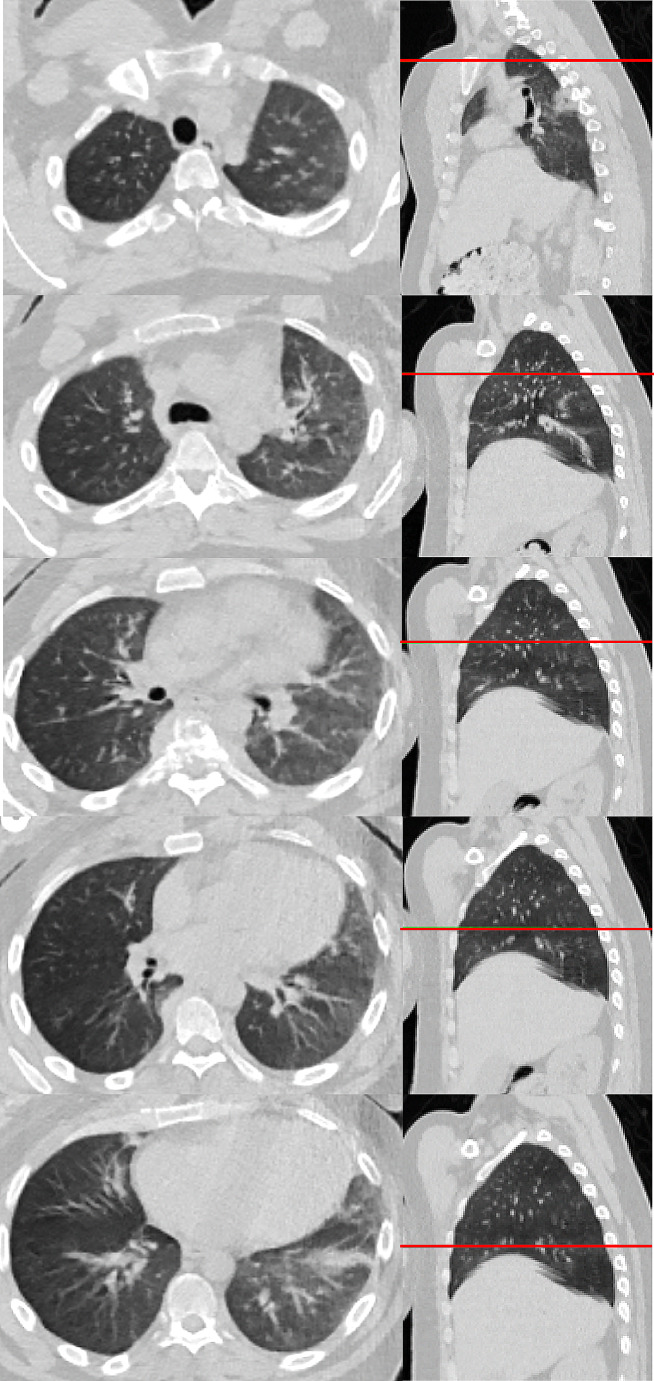
CT scan of the chest (lung-window) at different levels from cranial to caudal. Left side: axial view. Right side: saggital view with corresponding marking of level (red line)

The CT of the lungs did not show any TB-specific abnormalities or calcifications (Fig. 5).

### Further TB investigations

No *Mtb* was cultured using MGIT (Mycobacteria growth indicator tube) from the sputum sample after 42 days of incubation. However, we detected “trace” amount of bacillary load (cycle threshold 23.8) of *Mtb* in Xpert MTB/RIF Ultra of the sputum sample. After 14 days, the urine sample *Mtb* culture was positive, sensitive to Rifampicin and Isoniazid and “low” bacillary load (cycle threshold 17.9) was detected using Xpert MTB/RIF Ultra. After the initial PET/CT, the patient underwent a CT-guided biopsy for tissue confirmation of the working diagnosis and showed a negative culture for *Mtb* and no other bacterial growth was detected. However, Xpert MTB/RIF Ultra showed a positive, Rifampicin suseptible result for the spinal specimen (cycle threshold 27.5) and histological examination showed granulomatous inflammation with no AFB (acid fast bacilli) observed. (Table 3) Negative Mtb culture, but positive Xpert MTB/RIF Ultra is a common finding in this paucibacillary disease [13]. Due to the positive Xpert MTB/RIF Ultra result from the spinal biopsy and positive *Mtb* culture and Xpert MTB/RIF Ultra of the urine sample, the patient was diagnosed with non-contiguous multi-level STB and urogenital TB without pulmonary involvement, and the patient was started on weight-based standard TB treatment (weight: 88.2 kg; 750 mg Rifampicin, 375 mg Isoniazid, 2 g Pyrazinamide, 1375 mg Ethambutol) orally once daily.

**Table 3:** Microbiological and histological findings of sputum, urine and spinal tissue specimen

	GeneXpert	CT-value	MGIT	Days to positivity	Histology
**Sputum**	+	23.8	-	N/A	N/A
**Urine**	+	17.9	+	14	N/A
**Spinal tissue**	+	27.5	-	N/A	granulomatous inflammation

CT: cycle threshold

### 6-month follow-up

At this timepoint, the patient`s spinal pain had resolved (pVAS 0/10), neither neurological nor neurogenic symptoms, nor urogenital or constitutional symptoms present, and the antalgic gait had almost resolved. The patient reported full compliance with TB treatment and had gained more than 3 kg to 91.4 kg. CRP decreased from 51 mg/l to 15 mg/l and haemoglobin increased from 10.8 g/dl to 12.9 g/dL. (Table 1) The 6-month follow-up PET/CT showed new lesions of T10 and L5. Due to the significant clinical and laboratory improvement, no further actions were taken regarding the new lesions on PET/CT and the patient was discharged with continued TB treatment for a full treatment duration of 12 months.

### 9-month follow-up

Nine months after recruitment, the patient complained of acute urinary retention. Cauda equina syndrome as well as complicated urinary tract infection were ruled out and the patient was referred to the department of urology after insertion of a suprapubic catheter. The final diagnosis of a urogenital TB related urethral stricture was made.

### 12-month follow-up

At the last study PET/CT (12 months), the patient presented clinically cured with a normal gait, no resting or mechanical pain in the spinal column, absence of TB specific symptoms and good compliance with the suprapubic catheter. The patient reported full compliance with TB treatment. His weight decreased from 91.4 kg to 87.0 kg, which according to the patient was due to a more active lifestyle. CRP decreased from 15 mg/l to 12 mg/l and haemoglobin increased from 12.9 g/dL to 14.0 g/dl. (Table 1) The patient adhered to the full 12-month TB treatment and was discharged from our orthopaedic department and the patient completed the study as per protocol. The patient is currently awaiting urethroplasty.

### PET/CT scan reading

**Fig. 6:**
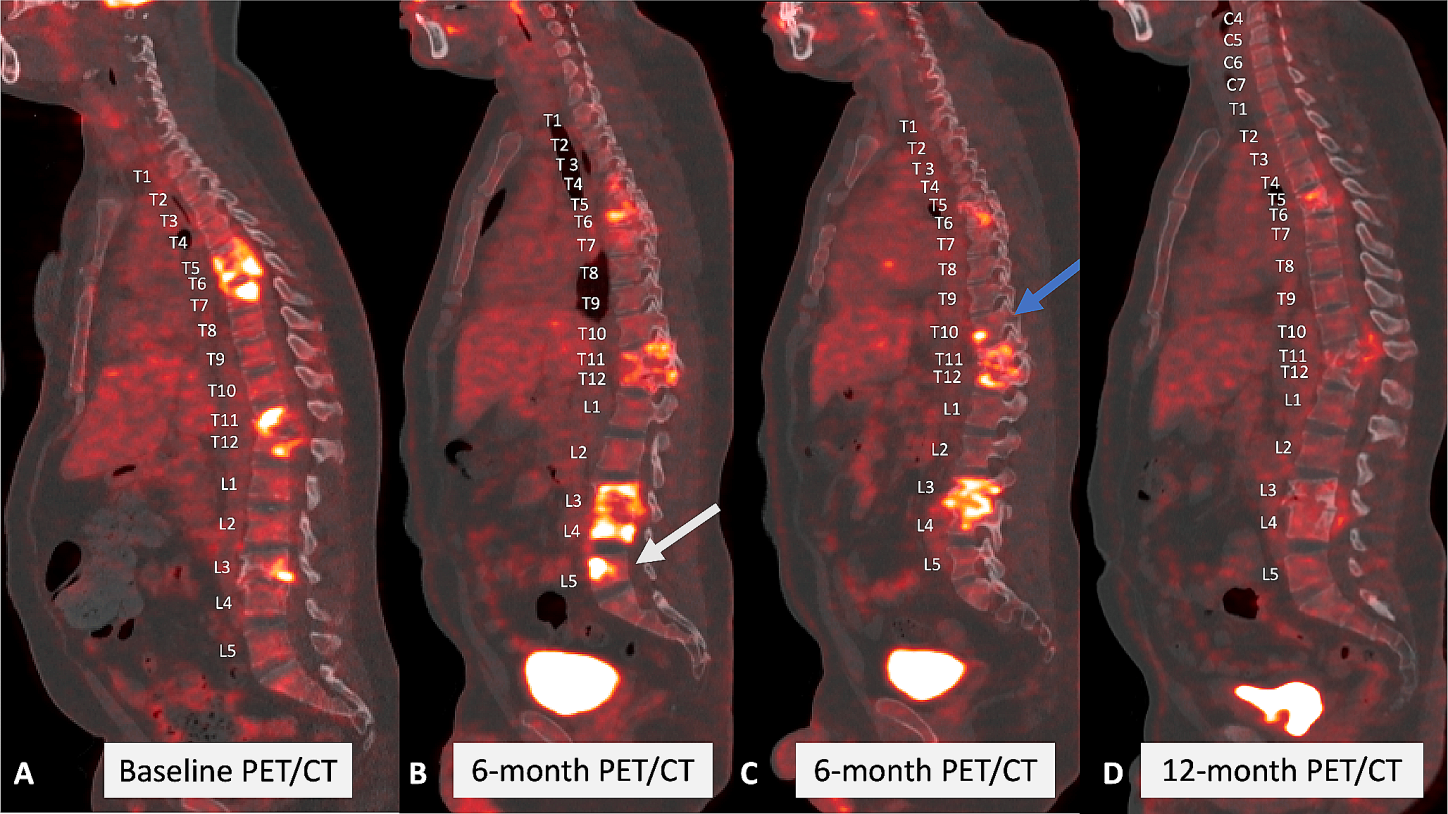
Comparison of initial, 6-months and 12-months follow-up spinal PET/CT (bone window) **A**: Sagittal view of initial PET/CT. **B**: Sagittal view of 6-months follow-up; white arrow: new L5 lesion. **C**: Sagittal view of 6-months follow-up; blue arrow: new Th10 lesion. **D**: Sagittal view of 12-months follow-up

**Fig. 7:**
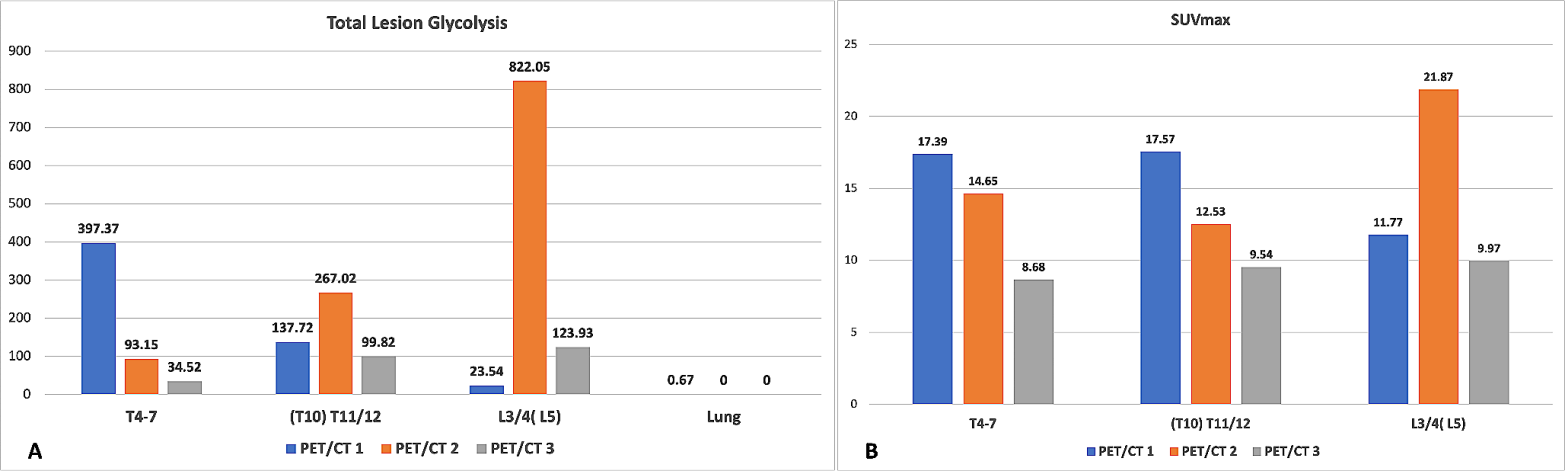
Bar chart of PET/CT values over time stratified by spinal lesions and lung **A**: Total lesion glycolysis over time. **B**: SUVmax over time (no lung value)

Spinal PET/CT reading was performed using MIM® Version 7.2.8 (MIM Software Inc., Cleveland, Ohio, USA). Maximum standardized uptake value (SUVmax) and mean standardized uptake value (SUVmean) as well as total lesion glycolysis (TLG) of spinal lesions were measured. Firstly, every not apparently diseased vertebra was assessed regarding its SUVmax. A sphere was placed in the central aspect of the vertebra with maximum radius including only cancellous bone. Values of all apparently not diseased vertebras were averaged, and two standard deviations (SD) were added to create a specific “healthy” SUV-value for calculating the pathological TLG of the region of interest (ROI). The rationale behind this approach is that TB is a systemic disease, resulting in a systemic inflammatory response commonly with concomitant anaemia and subsequent upregulation of bone marrow activity with increased SUV uptake, even in apparently non-diseased vertebras [14, 15]. Therefore, every apparent non-*Mtb* infected vertebra should still be considered diseased, and we aimed to record the TLG of lesions caused by *Mtb*-related inflammation only, without general background bone marrow abnormality. After determination of the subthreshold value, a ROI was drawn around each lesion and the patient-specific subthreshold was applied to measure SUVmax, SUVmean and TLG. Lung PET/CT reading was performed as previously described for the PredictTB trial [16]. Spinal PET/CT imaging at the different timepoints (initial, 6-month and 12-month) as well as PET/CT values of the spinal lesions and whole lung are depicted in Fig. 6 (spinal PET/CT), Table 4 (PET/CT values over time) as well as Fig. 7 (graphical depiction of PET/CT values over time).

**Table 4:** Comparison of SUV-parameters between PET/CTs

	PET/CT 1				PET/CT 2				PET/CT 3			
**Lesion**	*T4-7*	*T11/12*	*L3/4*	*Lung*	*T4-7*	T*10–12	L3-5*	*Lung*	*T4-7*	*T10-12*	*L3-5*	*Lung*
**TLG**	397.37	137.72	23.54	0	93.15	267.02	822.05	0	34.52	99.82	123.93	0
**SUVmax**	17.39	17.57	11.77	N/A	14.65	12.53	21.87	N/A	8.68	9.54	9.97	N/A
**SUVmean**	9.42	7.93	7.54	N/A	6.22	5.92	7.33	N/A	4.92	5.03	4.94	N/A
**Calculated subthreshold**	6.04			N/A	4.5			N/A	3.92			N/A

TLG: Total lesion glycolysis; Th: thoracic spine; L: lumbar spine; SUVmax: maximum standardized uptake value,

SUVmean: mean standardized uptake value; *: new lesion

Some spinal lesions showed an increase in SUVmax and TLG values at the 6-month PET/CT compared to the baseline PET/CT, but all spinal lesions decreased in both TLG and SUVmax at the 12-month PET/CT compared to the baseline PET/CT.

## Discussion and conclusion

We present the case of a young HIV-uninfected man diagnosed with non-contiguous multi-level spinal tuberculosis alongside concurrent urogenital TB, demonstrating self-reported adherence to TB treatment and demonstrating a favourable clinical response to treatment for drug-sensitive TB. Notably, during the 6-month follow-up, the PET/CT scan revealed the development of new spinal lesions and progression of existing lesions. This case warrants careful consideration of several key aspects.

### Paradoxical reaction in tuberculosis

The phenomenon of clinical and radiological deterioration of TB under appropriate TB treatment, termed “paradoxical reaction”, has been described for decades - most commonly in pulmonary TB. Additionally, in HIV-infected individuals a paradoxical worsening of TB symptoms has been described as immune reconstitution inflammatory syndrome (IRIS) in patients with pulmonary TB starting antiretroviral therapy, as well as TB meningitis and other forms of TB [17–23]. An increased host response to mycobacteria antigens have been linked to the development of a paradoxical reaction after treatment initiation [24]. However, our patient is HIV-uninfected and did not show any clinical deterioration despite two discrete new lesions at the 6-month follow-up PET/CT. A comprehensive review of the literature revealed only one case report and one case series with paradoxical reaction in primary pulmonary TB or TB meningitis resulting in new spinal lesions [25, 26]. A case series from 2015, reporting on four patients with paradoxical reaction, assessed three patients with worsening of the spinal lesions with concomitant pulmonary or miliary TB. Only one patient was reported as isolated STB (no pulmonary lesions) with worsening of the spinal lesion after TB treatment initiation [27]. This was an aggravation of a known spinal lesion identified by MRI. In our case, two discrete new lesions developed under appropriate TB treatment, which were not detected on the initial MRI or PET/CT, which, to the best of our knowledge, has not yet been described in literature. There are several possible explanations for this. Firstly, the newly developed lesions were below level of detection in the baseline dual imaging of PET/CT and MRI and increased on TB treatment, which is unlikely under adherent drug therapy. Secondly, the newly detected increased uptake on PET/CT at the 6-month follow-up might be due to healing of these previously not detected lesions. The phenomenon of increased SUV in sterile postsurgical healing stages has been linked to increased metabolic activity of inflammatory cells and osteo- and fibroblasts due to tissue and bone healing [28, 29]. Thirdly, the patient could be suffering from a dual infection with a drug-sensitive and a drug-resistant strain of which the drug-resistant strain was not detected in the spinal biopsy, which however seems unlikely considering the full clinical recovery of the patient at 12 months follow-up. Lastly, as described by Malherbe et al., PET/CT activity may be persistent or even increased after treatment completion in patients with pulmonary TB, indicating ongoing *Mtb* replication and insufficient sterilisation, which could explain the new lesions as well as the increased uptake identified at 6-month PET/CT and the persistent but significantly decrease of 18 F-FDG-uptake at the end of treatment PET/CT at 12 months [30]. There may be an increased probability for relapse in patients with persistent spinal inflation at the time of TB treatment completion and long-term cohorts are needed to study the risk factors of relapse and post-tuberculosis disease.

### Value of PET/CT in STB

To our knowledge, only one study has investigated the diagnostic value of MRI and PET/CT in imaging diagnosis of STB and showed that PET/CT was underperforming in the detection of spinal lesions compared to MRI, a finding that we were not able to confirm in the presented case [31]. The discordance between MRI and PET/CT might be explained by cold spinal abscesses and non FDG avid vertebral destruction which do not show increased uptake in the PET/CT but are distinctively detected by MRI. The patient`s immune status and thus cell-mediated immune response towards *Mtb* infection and the duration of active disease might have an impact on the avidity of spinal lesions on PET/CT [32]. 

Only a few studies have reported on PET/CT findings in STB with only four studies reporting on average SUVmax values amongst the assessed spinal lesions at the initial PET/CT ranging from 10.2 to 17.8 [33–40]. Go et al., reported an initial SUVmax in one patient of 17.4, but did not assess the SUVmax at the eight months follow-up PET/CT [37]. Rai et al. reported on 25 patients with STB and assessed a mean SUVmax decrease from 14.88 at the initial PET/CT to 9.08 at the 6 months follow up PET/CT, which is conflicting with our findings [38]. However, it must be noted that comparisons between different studies should be drawn carefully, since different scanners, different dosage injection protocols, as well as biological factors are known to increase variability between absolute uptake values and therefore, a standardized protocol should be applied for every patient and their follow-ups [41]. Another source of variability in the SUVmax of the spinal lesions between individuals could be the extent of the disease (e.g., additional pulmonary disease versus isolated STB) in patients with additional lesions “consuming” more of the injected FDG which leaves less FDG available for uptake in the spinal lesions. It has been shown that increased FDG uptake is not only a result of increased metabolic activity due to malignancy or infection but can also reflect tissue regeneration which could explain the increased uptake in our patient after six months of TB treatment [42]. 

Despite increasing PET/CT values at the six-month follow-up, there was a significant decrease in all spinal lesions at treatment completion compared to the initial PET/CT, which may indicate healing status if the patient is clinically cured [43]. However, concrete conclusions on the radiological “healed status” identified by PET/CT imaging cannot yet be drawn. Thus, in our Spinal TBX Cohort, we aim to further investigate the value of PET/CT compared to the “imaging gold standard” MRI as a new imaging modality for STB.

### Route of infection in STB

TB is spread primarily through air and is transmitted via inhalation of *Mtb* containing droplets. Approximately 5% of exposed individuals develop active TB within the first year after infection, while 95% of infected individuals develop a latent form of TB [44]. In some infected individuals a Ghon complex may be formed, which is characterized by the Ghon focus (primary lesion, often subpleural), local lymphangitis and enlarged local lymph nodes [45]. The Ghon complex may develop into the Ranke complex, which is characterized by a calcified Ghon complex and calcified mediastinal lymph nodes [46]. All these typical radiological findings in primary pulmonary TB are absent on PET/CT in our patient, however, *Mtb* was detected in sputum by Xpert MTB/RIF Ultra with “trace” result, whereas the MGIT culture showed no growth of *Mtb*. After decontamination of the sputum sample, only 10 bacillli/ml of sputum are required to produce positive culture results, which in our case either means that there was a very low bacillary burden, or the infection was cleared and the Xpert MTB/RIF Ultra only detected genetic material of dead *Mtb* bacilli [47]. The remaining question is how such an extensive *Mtb* infection of the spine can occur, without primary infection of the lung? Besides airborne transmission of *Mtb*, other ways of TB transmission (direct contact through blood or other body fluids) have been described [48]. Several studies have shown that most patients with STB have a primary lung focus or a history of pulmonary TB [49–52], however, our patient did not have a previous history of pulmonary TB. Further, it has been shown that *Mtb* can be transmitted via arterial or venous routes from a primary focus of the urogenital system to the spinal column, which may be the mode of dissemination in the presented patient [53].

## Conclusion

Conclusions: In our case, PET/CT emerged as a valuable imaging modality for the initial assessment, surpassing MRI by revealing more comprehensive extensive disease. Subsequent PET/CT scans at 6-month uncovered new lesions and increased inflammation in existing ones, while by the end of TB treatment, all lesions exhibited improvement. However, the interpretation of FDG avidity remains ambiguous, whether it correlates with active infection and viable Mtb. or fibro- and osteoblast activity indicative of the healing process. Additionally, the absence of extraspinal TB lesions on PET/CT despite positive microbiology from sputum and urine maybe explained by paucibacillary, subclinical infection of extraspinal organs. The Spinal TB X Cohort endeavours to shed light on whole-body imaging patterns at diagnosis, their evolution midway through TB treatment, and upon treatment completion. Ultimately, this study aims to advance our understanding of the biology of this complex disease.

## Data Availability

Data is available on reasonable request.
